# Significance of geriatric nutritional risk index in predicting lung-transplant waiting list mortality of patients with interstitial lung disease regardless of percentage forced vital capacity

**DOI:** 10.1007/s11748-026-02273-z

**Published:** 2026-02-28

**Authors:** Chihiro Konoeda, Gouji Toyokawa, Miho Yamaguchi, Takafumi Yamaya, Takaki Aakamine, Mitsuaki Kawashima, Mototsugu Shimokawa, Masaaki Sato

**Affiliations:** 1 Department of Thoracic Surgery, The University of Tokyo Hospital, 7-3-1 Hongo, Bunkyo-ku, Tokyo, 113-8655 Japan; 2 Department of Surgery and Science, Graduate School of Medical Sciences, Kyushu University, Fukuoka, Japan; 3 Department of Biostatistics, Graduate School of Medicine, Yamaguchi University, Yamaguchi, Japan

**Keywords:** Geriatric Nutritional Risk Index, Lung transplantation, Interstitial lung disease, Respiratory function

## Abstract

**Objective:**

The geriatric nutritional risk index (GNRI) is derived from serum albumin, current body weight, and ideal body weight. It is a prognostic indicator for waitlist mortality in patients awaiting lung transplantation (LT). However, its significance according to respiratory function in patients with interstitial lung disease (ILD) awaiting LT remains unclear.

**Methods:**

We retrospectively analyzed adult patients with ILD listed for LT from donation after brain death between January 2014 and July 2024. They were divided into two groups based on a cutoff of 50% for percentage forced vital capacity (%FVC). A GNRI cutoff value of 93.84 was established based on our previous study.

**Results:**

Among 253 patients, 123 (48.6%) underwent LT, 81 (32.0%) died while awaiting LT, and 49 (19.4%) remained on the waiting list. The high- and low-%FVC groups included 123 (48.6%) and 130 patients (51.4%), respectively. In the high-%FVC group, 105 (85.4%) and 18 (14.6%) patients had a high and low GNRI, respectively. In the low-%FVC group, 78 (60.0%) and 52 (40.0%) patients had a high and low GNRI, respectively. In both groups, patients with a low GNRI had significantly shorter survival compared with those having a high GNRI (both *P* = 0.001). In multivariate analyses, low GNRI was an independent predictor of poor prognosis in both the high- and low-%FVC groups (hazard ratio = 4.918 and 2.576, *P* < 0.001 and = 0.001, respectively).

**Conclusions:**

The GNRI may serve as a prognostic factor for waitlist mortality in patients with ILD awaiting LT, regardless of %FVC.

**Supplementary Information:**

The online version contains supplementary material available at 10.1007/s11748-026-02273-z.

## Introduction

Lung transplantation (LT) is a life-saving treatment for patients with progressive end-stage respiratory diseases that do not respond to optimal medical therapies [1]. In Japan, the scarcity of donors and ‘‘first come, first served’’ principle for LT lead to a longer waiting time of approximately 700 days and a higher mortality rate of 33.1%, in comparison with Western countries [2]. Given that malnutrition and frailty are key indicators of waitlist mortality and post-transplant survival in lung transplant candidates, it is essential to manage factors that negatively affect the survival of patients during the waiting period for LT [3, 4]. Notably, among patients listed for LT, those with interstitial lung disease (ILD) exhibit poorer survival after being listed for LT [5]. Therefore, it is crucial to maintain transplant candidacy and reduce waitlist mortality, particularly for patients with ILD.

Several biomarkers have been assessed for their ability to predict waitlist mortality in patients awaiting LT. Notably in Japan, markers such as the prognostic nutritional index (PNI)—derived from serum albumin and lymphocyte count—and muscle mass have been reported to have an impact on waitlist mortality prior to LT [6–8]. Importantly, these markers are also useful in predicting postoperative outcomes following LT [9, 10]. This evidence underscores the importance of maintaining nutrition and muscle mass to reduce waitlist mortality and improve postoperative survival after LT. Recently, we showed that the geriatric nutritional risk index (GNRI)—derived from serum albumin, patient body weight, and ideal body weight [11]—may be a good prognostic indicator for waitlist mortality in adult patients listed for LT from donation after brain death (DBD) [12].

Percentage forced vital capacity (%FVC) is an essential determinant for the timing of referral to LT institutions, with 80% as the recommended cutoff for referral [13]. Additionally, %FVC is a component of the ILD-GAP (gender, age, physiology) score, which accurately predicts mortality in patients with ILD, with cutoffs of 50% and 75% [14]. A recent study demonstrated the prognostic utility of the ILD-GAP score in patients with ILD evaluated for LT: patients with higher ILD-GAP scores were significantly associated with shorter survival during the waiting period [15]. However, the importance of nutritional factors, such as the GNRI, remains unclear for patients with ILD awaiting LT according to the %FVC or ILD-GAP score.

Since lymphocyte count, a component of PNI, can be strongly affected by multiple conditions relatively common among patients with ILD, such as steroid use, infection, and inflammation, we hypothesized that GNRI might be superior to PNI in reflecting patients’ nutritional status and predicting waitlist mortality in this population. In the present study, we aimed to analyze the associations of the GNRI with patient characteristics and waitlist mortality in patients with ILD listed for LT from DBD, according to their %FVC with a cutoff of 50% based on the ILD-GAP score.

## Methods

### Study cohort

Between January 2014 and July 2024, a total of 478 adult patients aged 18 years or older were listed for LT from DBD at The University of Tokyo Hospital. Among them, 253 (52.9%) patients were diagnosed with ILD. These patients were divided into two groups based on a cutoff of 50% for %FVC, in accordance with the threshold of %FVC used in the ILD-GAP score [14]. We retrospectively obtained patients’ data at the time of being listed for LT from DBD in the Japan Organ Transplant Network. Data regarding LT from DBD, waiting time, and mortality were collected until the cut-off date in July 2025.

### Calculation of the GNRI and its cut-off value

Blood tests were performed when patients were admitted to our hospital to assess their eligibility for listing for LT. The cut-off value of the GNRI, calculated using the following formula: (1.489 × serum albumin [g/dL]) + (41.7 x [current body weight/ideal body weight]) [11], was set as 93.84 based on our previous study [12].

### Statistical analysis

Categorical variables are summarized as number and percentage, and continuous variables are presented as the median with first and third interquartile range (IQR). Overall survival (OS) was calculated from the date of listing in the Japan Organ Transplant Network for LT from DBD up to the date of death from any cause. Patients who underwent LT were censored at the time of the operation. The associations between the GNRI and continuous data were analyzed using the Student’s t-test for normally distributed data, and the Mann–Whitney or Kruskal–Wallis test for non-normally distributed data. The chi-squared test was used to analyze associations between the GNRI and categorical variables. Survival probabilities were estimated using the Kaplan–Meier method, and differences in the survival probabilities were analyzed using the log-rank test. Risk factors for waitlist mortality were assessed using a Cox proportional hazards model forced entry. Missing data were not complemented. Differences were considered to be statistically significant with a *P*-value < 0.05. All analyses were performed using JMP® 18.0 (SAS Institute, Cary, NC, USA) and Prism 8.0 (GraphPad Software, San Diego, CA, USA) software.

## Results

### Patient characteristics and study flow

The characteristics of 253 patients at the time of listing for LT from DBD are shown in **Supplementary Table 1**. The median age was 53 years (first, third IQR: 45, 57). There were 161 (63.6%) male patients, and 159 (62.8%) patients had a history of smoking. The median body mass index (BMI) and 6-min walk distance values were 22.2 kg/m^2^ (first, third IQR: 18.4, 25.7), and 340 m (first, third IQR: 270, 425), respectively. The breakdown of disease categories was as follows: idiopathic pulmonary fibrosis (IPF) (n = 58, 22.9%), pleuroparenchymal fibroelastosis (n = 36, 14.2%), and collagen tissue disease-associated interstitial pneumonia (n = 67, 26.5%). Oxygen supplementation at rest was required for 117 (46.2%) patients, and 145 (57.3%) patients had a history of treatment with steroids. Diffusing capacity of the lung for carbon monoxide (DLCO) data were missing for 40 patients. The median %FVC and GNRI were 49.6 (first, third IQR: 41.4, 61.0) and 101.45 (first, third IQR: 92.95, 109.39), respectively, and their association was weak (R^2^ = 0.1748; Y = 0.2653*X + 87.32; **Supplementary Fig. 1**).

The study flow diagram is shown in **Supplementary Fig. 2**. A %FVC cutoff of 50% was used to stratify waitlist mortality in a statistically significant manner (*P* = 0.038; **Supplementary Fig. 3**). The high- and low-%FVC groups included 123 (48.6%) and 130 patients (51.4%), respectively. In the high-%FVC group, 64 (52.0%) patients underwent LT, 33 (26.8%) patients died while awaiting LT, and 26 (21.2%) patients remained on the waiting list. In the low-%FVC group, 59 (45.4%) patients underwent LT, 48 (36.9%) patients died while awaiting LT, and 23 (17.7%) patients remained on the waiting list. In the high-%FVC group, 105 (85.4%) and 18 (14.6%) patients had a high and low GNRI whereas in the low-%FVC group, 78 (60.0%) and 52 (40.0%) patients had a high and low GNRI, respectively.

### Association between the GNRI and patient characteristics in patients with %FVC ≥ 50%

We divided the 123 patients with %FVC ≥ 50% into a %FVC ≥ 50%/GNRI-low group (n = 18, 14.6%) and %FVC ≥ 50%/GNRI-high group (n = 105, 85.5%) based on the cut-off value of the GNRI. The %FVC ≥ 50%/GNRI-low group had a lower BMI (*P* < 0.001), more disease categories other than IPF (*P* = 0.012), a lower forced expiratory volume in 1 s (FEV1.0; *P* = 0.012), a lower percentage DLCO (%DLCO)/alveolar volume (VA; *P* = 0.042), and higher partial pressure of carbon dioxide (pCO_2_; *P* = 0.010), as compared with the %FVC ≥ 50%/GNRI-high group (Table 1).

**Table 1:** Characteristics of the %FVC ≥ 50% / GNRI-low and %FVC ≥ 50%/GNRI-high groups at the time of registration for lung transplantation from donation after brain death

Demographics		%FVC ≥ 50%/GNRI-low group (n = 18 [14.6%])	%FVC ≥ 50%/GNRI-high group (n = 105 [85.5%])	*P-*value
Age (years), median (IQR)		56 (50, 58)	55 (50, 59)	0.923
Sex	Female	9 (50.0%)	30 (28.6%)	0.071
	Male	9 (50.0%)	75 (71.4%)	
Smoking history	Never	8 (44.4%)	29 (27.6%)	0.150
	Former	10 (55.6%)	76 (72.4%)	
BMI (kg/m^2^), median (IQR)		17.8 (16.6, 20.2)	24.3 (22.1, 29.0)	< 0.001
Blood type	A	6 (33.3%)	42 (40.0%)	0.804
	O	3 (16.7%)	18 (17.2%)	
	B	8 (44.4%)	35 (33.3%)	
	AB	1 (5.6%)	10 (9.5%)	
Type of fibrotic lung disease	IPF	1 (5.6%)	37 (35.2%)	0.012
	Others	17 (94.4%)	68 (64.8%)	
6-min walk distance (m), median (range)		315 (268, 402)	380 (292, 450)	0.220
Oxygen supplementation at rest	No	8 (44.4%)	65 (61.9%)	0.164
	Yes	10 (55.6%)	40 (38.1%)	
Pulmonary hypertension	No	15 (83.3%)	88 (83.8%)	0.960
	Yes	3 (16.7%)	17 (16.2%)	
Use of steroid	No	10 (55.6%)	41 (39.1%)	0.189
	Yes	8 (44.4%)	64 (60.9%)	
Use of antifibrotic agents	No	7 (38.9%)	35 (33.3%)	0.646
	Yes	11 (61.1%)	70 (66.7%)	
FEV1.0 (L), median (IQR)		1.6 (1.5, 2.0)	2.1 (1.8, 2.3)	0.012
%DLCO (%), median (IQR)*		35.3 (31.1, 43.3)	41.7 (32.2, 54.6)	0.146
%DLCO/VA (%), median (IQR)*		55.6 (43.0, 63.3)	74.0 (56.0, 84.9)	0.042
pO_2_ (mmHg), median (IQR)		72.6 (56.7, 90.5)	77.6 (68.9, 89.2)	0.235
pCO_2_ (mmHg), median (IQR)		44.2 (39.6, 48.3)	40.0 (37.4, 43.4)	0.010

BMI, body mass index; DLCO, diffusing capacity of the lung for carbon monoxide; FEV1.0, forced expiratory volume in 1 s; GNRI, geriatric nutritional risk index; IPF, idiopathic pulmonary fibrosis; IQR, interquartile range; pCO_2_, partial pressure of carbon dioxide; pO_2_, partial pressure of oxygen; %FVC, percentage forced vital capacity; VA, alveolar volume

^*^Data were missing for six patients

### Association between the GNRI and patient characteristics in patients with %FVC < 50%

We divided the 130 patients with %FVC < 50% into two groups: a %FVC < 50%/GNRI-low (n = 52, 40.0%) group and %FVC < 50%/GNRI-high (n = 78, 60.0%) group, based on the cut-off value of the GNRI. The %FVC < 50%/GNRI-low group had a lower BMI (*P* < 0.001), a higher frequency of pulmonary hypertension at listing (*P* = 0.007), a lower frequency of use of anti-interstitial agents (*P* < 0.001), a lower FEV1.0 (*P* < 0.001), a lower %DLCO/VA (*P* = 0.001), and a higher pCO_2_ (*P* < 0.001) compared with the %FVC < 50%/GNRI-high group (Table 2).

**Table 2:** Characteristics of the %FVC < 50% / GNRI-low and %FVC < 50% / GNRI-high groups at the time of registration for lung transplantation from donation after brain death

Demographics		%FVC < 50%/GNRI-low group (n = 52 [40.0%])	%FVC < 50%/GNRI-high group (n = 78 [60.0%])	*P-*value
Age (years), median (IQR)		49 (43, 55)	49 (42, 54)	0.597
Sex	Female	22 (42.3%)	31 (39.7%)	0.771
	Male	30 (57.7%)	47 (60.3%)	
Smoking history	Never	26 (50.0%)	31 (39.7%)	0.248
	Former	26 (50.0%)	47 (60.3%)	
BMI (kg/m^2^), median (IQR)		16.4 (15.8, 18.4)	22.8 (20.3, 26.6)	< 0.001
Blood type	A	19 (36.5%)	33 (42.3%)	0.539
	O	21 (40.4%)	22 (28.2%)	
	B	8 (15.4%)	16 (20.5%)	
	AB	4 (7.7%)	7 (9.0%)	
Type of fibrotic lung disease	IPF	5 (9.6%)	15 (19.2%)	0.137
	Others	47 (90.4%)	63 (80.8%)	
6-min walk distance (m), median (range)		300 (205, 395)	334 (240, 421)	0.133
Oxygen supplementation at rest	No	23 (44.2%)	40 (51.3%)	0.431
	Yes	29 (55.8%)	38 (48.7%)	
Pulmonary hypertension	No	35 (67.3%)	68 (87.2%)	0.007
	Yes	17 (32.7%)	10 (12.8%)	
Use of steroid	No	25 (48.1%)	32 (41.0%)	0.427
	Yes	27 (51.9%)	46 (59.0%)	
Use of antifibrotic agents	No	34 (65.4%)	27 (34.6%)	< 0.001
	Yes	18 (34.6%)	51 (65.4%)	
FEV1.0 (L), median (IQR)		1.1 (0.8, 1.3)	1.3 (1.1, 1.7)	< 0.001
%DLCO (%), median (IQR)*		38.1 (29.3, 55.5)	40.0 (31.2, 49.3)	0.706
%DLCO/VA (%), median (IQR)*		62.5 (51.6, 86.2)	86.3 (64.8, 104.6)	0.001
pO_2_ (mmHg), median (IQR)		75.0 (65.5, 85.6)	75.1 (65.0, 88.0)	0.860
pCO_2_ (mmHg), median (IQR)		46.1 (42.9, 54.2)	40.7 (37.9, 43.2)	< 0.001

BMI, body mass index; DLCO, diffusing capacity of the lung for carbon monoxide; FEV1.0, forced expiratory volume in 1 s; GNRI, geriatric nutritional risk index; IPF, idiopathic pulmonary fibrosis; IQR, interquartile range; pCO_2_, partial pressure of carbon dioxide; pO_2_, partial pressure of oxygen; %FVC, percentage forced vital capacity; VA, alveolar volume

^*^Data were missing for 34 patients

### Analysis of the risk factors for waitlist mortality

**Fig. 1:**
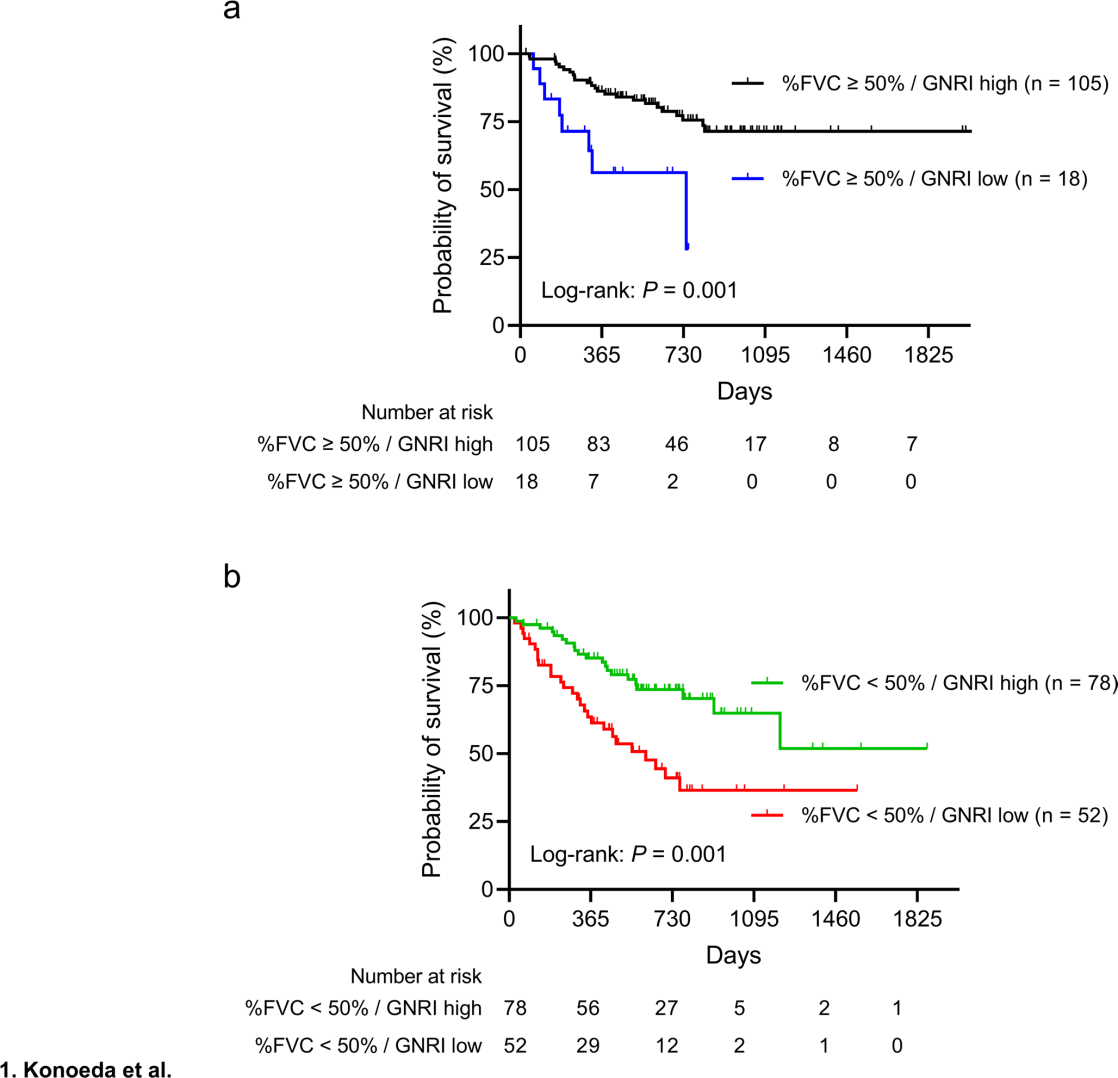
Waitlist mortality in 123 patients with %FVC ≥ 50% and 130 patients with %FVC < 50% listed for LT based on their GNRI. **a** The %FVC ≥ 50% group: low GNRI (< 93.84; n = 18) versus high GNRI (≥ 93.84; n = 105); log-rank: *P* = 0.001. **b** The %FVC < 50% group: low GNRI (< 93.84; n = 52) versus high GNRI (≥ 93.84; n = 78); log-rank, *P* = 0.001. GNRI, geriatric nutritional risk index; LT, lung transplantation; %FVC, percentage forced vital capacity

The median follow-up period was 562 days (first, third IQR: 318, 806). The %FVC ≥ 50%/GNRI-low group had a significantly shorter median survival time than the %FVC ≥ 50%/GNRI-high group (742 days vs. not reached; *P* = 0.001; Fig. 1a). Similarly, the %FVC < 50% / GNRI-low group had a significantly shorter median survival time than the %FVC < 50%/GNRI-high group (611 days vs. not reached; *P* = 0.001; Fig. 1b). In the entire population of 253 patients, a multivariate analysis using eight variables—including age, sex, smoking history, 6-min walk distance, oxygen supplementation at rest, %FVC, GNRI, and PNI—showed that GNRI (hazard ratio [HR], 2.826; 95% confidence interval [CI], 1.655–4.826; *P* < 0.001) and the 6-min walk distance (HR, 2.635; 95%CI, 1.577–4.403; *P* < 0.001), but not PNI (HR, 1.567; 95%CI, 0.935–2.625; *P* = 0.088), were independent prognostic factors for waitlist mortality. Multivariate analysis identified significant independent prognostic factors for survival during the waiting period in patients with %FVC ≥ 50%: sex (HR, 2.755; 95%CI, 1.102–6.887; *P* = 0.030), 6-min walk distance (HR, 3.467; 95%CI, 1.606–7.483; *P* = 0.002), and the GNRI (HR, 4.918; 95%CI, 2.064–11.716; *P* < 0.001; Table 3). For patients with %FVC < 50%, independent prognostic factors for survival during the waiting period included 6-min walk distance (HR, 1.890; 95%CI, 1.040–3.435; *P* = 0.037), and the GNRI (HR, 2.576; 95% CI, 1.441–4.606; *P* = 0.001).

**Table 3:** Results of multivariate analyses for waitlist mortality in patients waiting for lung transplantation according to %FVC

Variables	%FVC ≥ 50%			%FVC < 50%		
	HR	95% CI	*P* value	HR	95%CI	*P* value
Age (≥ 55 years/ < 55 years)	1.315	0.634–2.728	0.462	1.521	0.798–2.897	0.202
Sex (male/female)	2..755	1.102–6.887	0.030	1.248	0.681–2.285	0.474
6-min walk distance (< 250 m/ ≥ 250 m)	3.467	1.606–7.483	0.002	1.890	1.040–3.435	0.037
GNRI (low [< 93.84]/high [≥ 93.84])	4.918	2.064–11.716	< 0.001	2.576	1.441–4.606	0.001

CI, confidence interval; GNRI, geriatric nutritional risk index; HR, hazard ratio; %FVC, percentage forced vital capacity

## Discussion

This study showed that the GNRI was an independent prognostic factor in patients with ILD awaiting LT from DBD, regardless of %FVC. Our findings emphasize the importance of maintaining or improving nutritional status to decrease waitlist mortality, independent of %FVC. Clinicians may need to consider LT from marginal donors or living donors more actively for patients with a low GNRI, even when the %FVC exceeds 50%. In Western countries, lung allocation systems like the Composite Allocation Score are commonly used to select patients for LT. However, in countries like Japan, where donor numbers are low, the GNRI could also serve as an allocation marker for LT.

A low GNRI was significantly associated with a lower FEV1.0, lower %DLCO/VA, and higher pCO_2_, in comparison with a high GNRI in both the %FVC ≥ 50% and %FVC < 50% groups. Additionally, the %FVC < 50%/GNRI-low group had a higher frequency of pulmonary hypertension at listing compared with the %FVC < 50%/GNRI-high group. These findings indicate that the GNRI may serve as a surrogate for patients’ general status and disease progression, especially in the %FVC < 50% group. The lower frequency of anti-interstitial agent use, which can inhibit the progression of some types of ILD [16], in the %FVC < 50%/GNRI-low group versus the %FVC < 50%/GNRI-high group also supports the potential of the GNRI as a surrogate for disease progression. Regarding why a low GNRI was more prevalent in non-IPF ILDs in patients with %FVC ≥ 50%, patients with PPFE was more frequently included in the low GNRI group compared to the high GNRI group (33.3% [6/18] vs. 3.8% [4/105]). Generally, the body weight of patients with PPFE is lower than that of those with other ILDs [17]. Our data also showed that among 123 patients with %FVC ≥ 50%, the median body weight in patients with PPFE was lower than that in those with IPF, CTD-ILD, and others (50.5 kg, 69.9 kg, 56.1 kg and 67.8 kg, respectively; *P* < 0.001). These findings partially support the result that a low GNRI was more prevalent in non-IPF ILD with %FVC ≥ 50%.

Previous reports have indicated that several markers might serve as prognostic indicators for waitlist mortality in LT candidates. The PNI, calculated from serum albumin and lymphocyte count, has been shown to be a significant predictor of waitlist mortality with a cut-off value of 45.8 [6]. In our cohort, using this cut-off value, among 123 patients with %FVC ≥ 50%, those with a low PNI exhibited significantly lower survival than those who had a high PNI (*P* = 0.001; **Supplementary Fig. 4a**). By contrast, among 130 patients with %FVC < 50%, survival did not differ significantly between patients with high and low PNI (*P* = 0.278; **Supplementary Fig. 4b**). This might be because body weight is more important than lymphocyte count in this population, although this is speculative. Since lymphocyte count, a component of PNI, can be strongly affected by steroid use, infection, and inflammation in ILD patients, PNI might not accurately reflect patients’ immune and nutritional status. In fact, 73 patients (56.2%) among the 130 patients with %FVC < 50% received steroids. These findings suggest that the GNRI might be superior to the PNI in predicting waitlist mortality among patients with ILD listed for LT, regardless of %FVC, even though both indices are inexpensive and readily available. Additionally, the prognostic significance of psoas muscle mass in waitlist mortality for LT has been reported [7]; however, measuring muscle mass requires special imaging modalities that cannot be easily applied in clinical settings. By contrast, the GNRI is easily calculated using serum albumin, patient body weight, and ideal body weight, without the need for special diagnostic tests, suggesting that the GNRI can be readily applied in clinical settings.

Our findings also highlight the potential usefulness of nutritional intervention based on the GNRI at listing for LT to reduce waitlist mortality and maintain transplant candidacy, especially in countries like Japan, where the waiting period is long. Although methods to improve nutrition during the waiting period for LT are yet to be established, dietary education for patients and their families, along with nutritional supplements, currently serve as the primary approach to improving nutritional status for LT candidates. Specific medical treatments to enhance nutritional status are required to reduce waitlist mortality and maintain transplant candidacy. We are currently conducting an exploratory trial on the inhibitory effect of anamorelin [18]—a ghrelin-receptor agonist approved for the treatment of cachexia characterized by weight loss in certain types of cancer—on body weight loss in patients with idiopathic pleuroparenchymal fibroelastosis awaiting LT (clinical trial number: jRCTs031250402). This clinical trial aims to clarify the impact of nutritional intervention on waitlist mortality and transplant candidacy in such patients.

The present study has several limitations. First, this was a retrospective study conducted at a single institution. Second, although 253 patients were included, the number of patients in the %FVC ≥ 50%/GNRI-low group was small (n = 18). Third, there were 33 deaths in the %FVC ≥ 50% group, resulting in a limited number of variables included in the multivariate analysis. Fourth, the impact of the GNRI on post-transplant outcomes was not assessed in the current study. Fifth, it is unclear whether measuring serum albumin at listing for LT is the best timing for predicting waitlist mortality. Sixth, we did not investigate the impact of the GNRI in relation to the decline in %FVC, which is an important factor for the timing of referral to LT institutions and listing for LT [13]. Seventh, our study lacked DLCO data for 40 patients; furthermore, all included patients were aged 60 years or younger owing to Japanese law regarding LT listing, which limits the accurate stratification of patients using the ILD-GAP score [14]. Eighth, the present study used a dichotomized approach, and did not use continuous modeling of %FVC and GNRI, which could enhance individualized risk prediction and support future score development. Future large studies with more patients are warranted to address these limitations.

In conclusion, the GNRI was shown to be a prognostic factor for waitlist mortality in patients with ILD awaiting LT, regardless of %FVC. The GNRI may serve as a useful marker for identifying patients at greater risk of waitlist mortality. Such patients might benefit from nutritional intervention to reduce waitlist mortality and maintain transplant candidacy. 

## Supplementary Information

Below is the link to the electronic supplementary material.

Supplementary Material 1

Supplementary Figure 1. Association between %FVC and GNRI in 253 patients with ILD. ILD: interstitial lung disease; %FVC, percentage forced vital capacity; PNI, prognostic nutritional index

Supplementary Figure 2. Study flowchart. The analyzed population comprised 253 adult patients with ILD listed for LT from DBD according to %FVC. DBD, donation after brain death; ILD, interstitial lung disease; LT, lung transplantation; %FVC, percentage forced vital capacity

Supplementary Figure 3. Waitlist mortality in 123 patients with %FVC ≥ 50% and 130 patients with %FVC < 50% listed for LT. The %FVC ≥ 50% group versus %FVC < 50%; log-rank: P = 0.038. LT, lung transplantation; %FVC, percentage forced vital capacity

Waitlist mortality in 123 patients with %FVC ≥ 50% and 130 patients with %FVC < 50% listed for LT based on their PNI. (a) The %FVC ≥ 50% group: low PNI (< 45.8; n = 53) versus high PNI (≥ 45.8; n = 70); log-rank: P = 0.001. (b) The %FVC < 50% group: low PNI (< 45.8; n = 74) versus high PNI (≥ 45.8; n = 56); log-rank: P = 0.278. LT, lung transplantation; %FVC, percentage forced vital capacity; PNI, prognostic nutritional index

## Data Availability

The data underlying this article will be shared on reasonable request to the corresponding author.
