# Supplementary material for: Significance of geriatric nutritional risk index in predicting lung-transplant waiting list mortality of patients with interstitial lung disease regardless of percentage forced vital capacity
Source: Gen Thorac Cardiovasc Surg. 2026 Feb 28;74(7):702–9. doi: 10.1007/s11748-026-02273-z (PMC13283179; doi:10.1007/s11748-026-02273-z)
Supplement: Supplementary file 1 — Supplementary Material 1 [file 11748_2026_2273_MOESM1_ESM.docx]

**Supplementary Table 1.** Characteristics of 253 adult patients with interstitial lung disease at the time of registration for lung transplantation from donation from brain death.

| **Demographics** |  | **N (%) or median value (range)** |
| --- | --- | --- |
| Age at registration (years) | Median (IQR) | 53 (45, 57) |
| Sex | Female | 92 (36.4%) |
|  | Male | 161 (63.6%) |
| Smoking history | Never | 94 (37.2%) |
|  | Former | 159 (62.8%) |
| BMI (kg/m^2^) | Median (IQR) | 22.2 (18.4, 25.7) |
| 6-minute walk distance (m) | Median (IQR) | 340 (270, 425) |
| Blood type | A | 100 (39.5%) |
|  | O | 64 (25.3%) |
|  | B | 67 (26.5%) |
|  | AB | 22 (8.7%) |
| Type of ILD | IPF | 58 (22.9%) |
|  | PPFE | 36 (14.2%) |
|  | Collagen tissue disease-associated | 67 (26.5%) |
|  | Other | 92 (36.4%) |
| Oxygen supplementation at rest |  | 117 (46.2%) |
| Pulmonary hypertension |  | 47 (18.6%) |
| Use of steroid |  | 145 (57.3%) |
| Use of antifibrotic agents |  | 150 (59.3%) |
| FVC (L) | Median (IQR) | 1.8 (1.3, 2.3) |
| %FVC (%) | Median (IQR) | 49.6 (41.4, 61.0) |
| FEV1.0 (L) | Median (IQR) | 1.6 (1.2, 2.1) |
| %DLCO (%)* | Median (IQR) | 40.4 (31.2, 53.0) |
| %DLCO/VA (%)* | Median (IQR) | 75.0 (58.2, 89.0) |
| pO_2_ (mmHg) | Median (IQR) | 76.6 (65.8, 87.0) |
| pCO_2_ (mmHg) | Median (IQR) | 41.4 (38.0, 45.4) |
| Albumin (g/dL) | Median (IQR) | 4.0 (3.7, 4.2) |
| GNRI | Median (IQR) | 101.45 (92.95, 109.39) |

BMI, body mass index; DLCO, diffusing capacity of the lung for carbon monoxide; FEV1.0, forced expiratory volume in 1 second; FVC, forced vital capacity; GNRI, geriatric nutritional risk index; ILD, interstitial lung disease; IPF, idiopathic pulmonary fibrosis; IQR, interquartile range; pCO_2_, partial pressure of carbon dioxide; pO_2_, partial pressure of oxygen; PPFE, pleuroparenchymal fibroelastosis; VA, alveolar volume.

*Data were missing for 40 patients
