# Supplementary figures and images for: Significance of geriatric nutritional risk index in predicting lung-transplant waiting list mortality of patients with interstitial lung disease regardless of percentage forced vital capacity
Source: Gen Thorac Cardiovasc Surg. 2026 Feb 28;74(7):702–9. doi: 10.1007/s11748-026-02273-z (PMC13283179; doi:10.1007/s11748-026-02273-z)

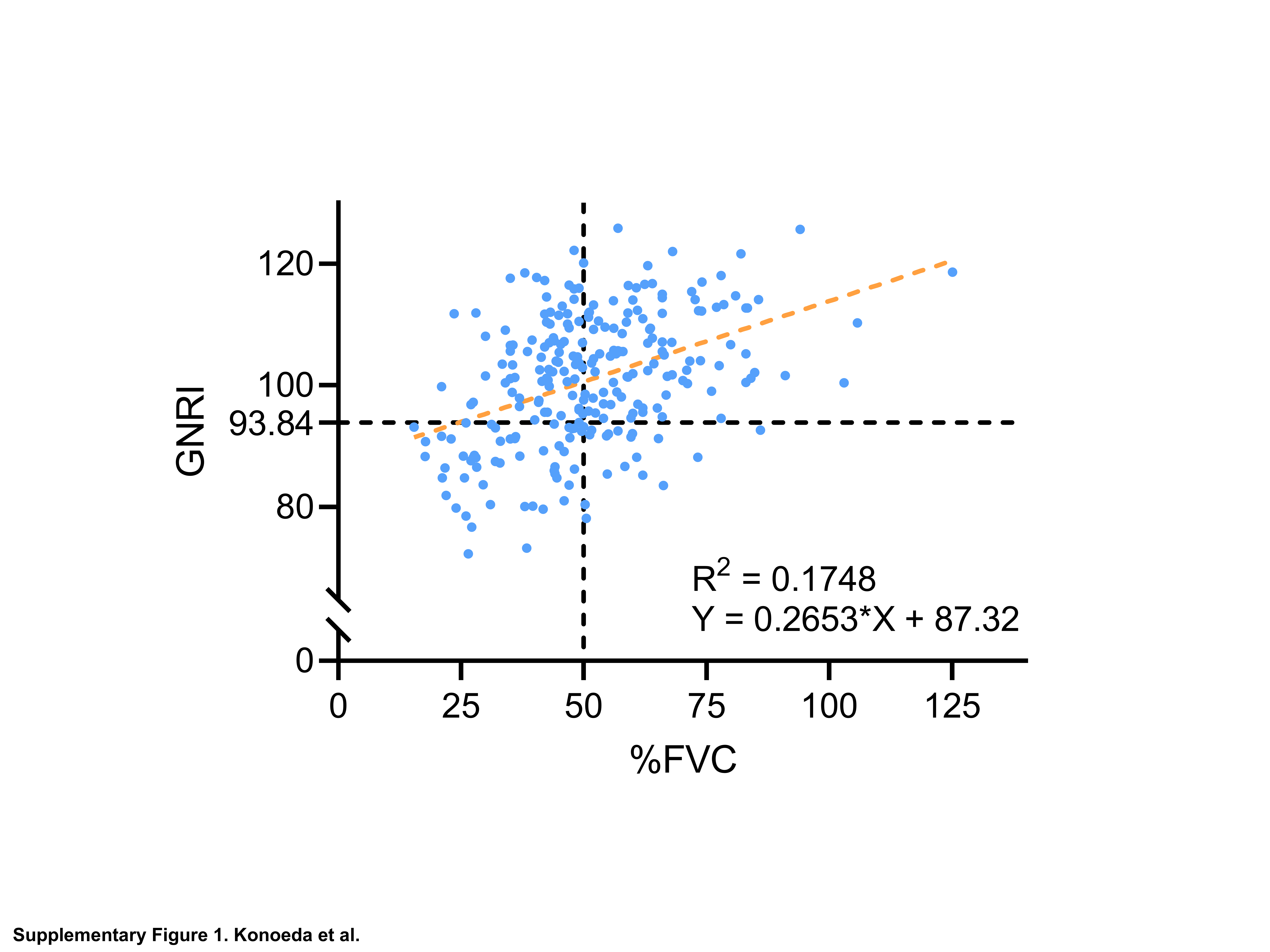

Supplement: Supplementary file 2 — Supplementary Figure 1. Association between %FVC and GNRI in 253 patients with ILD. ILD: interstitial lung disease; %FVC, percentage forced vital capacity; PNI, prognostic nutritional index [file 11748_2026_2273_MOESM2_ESM.tif]

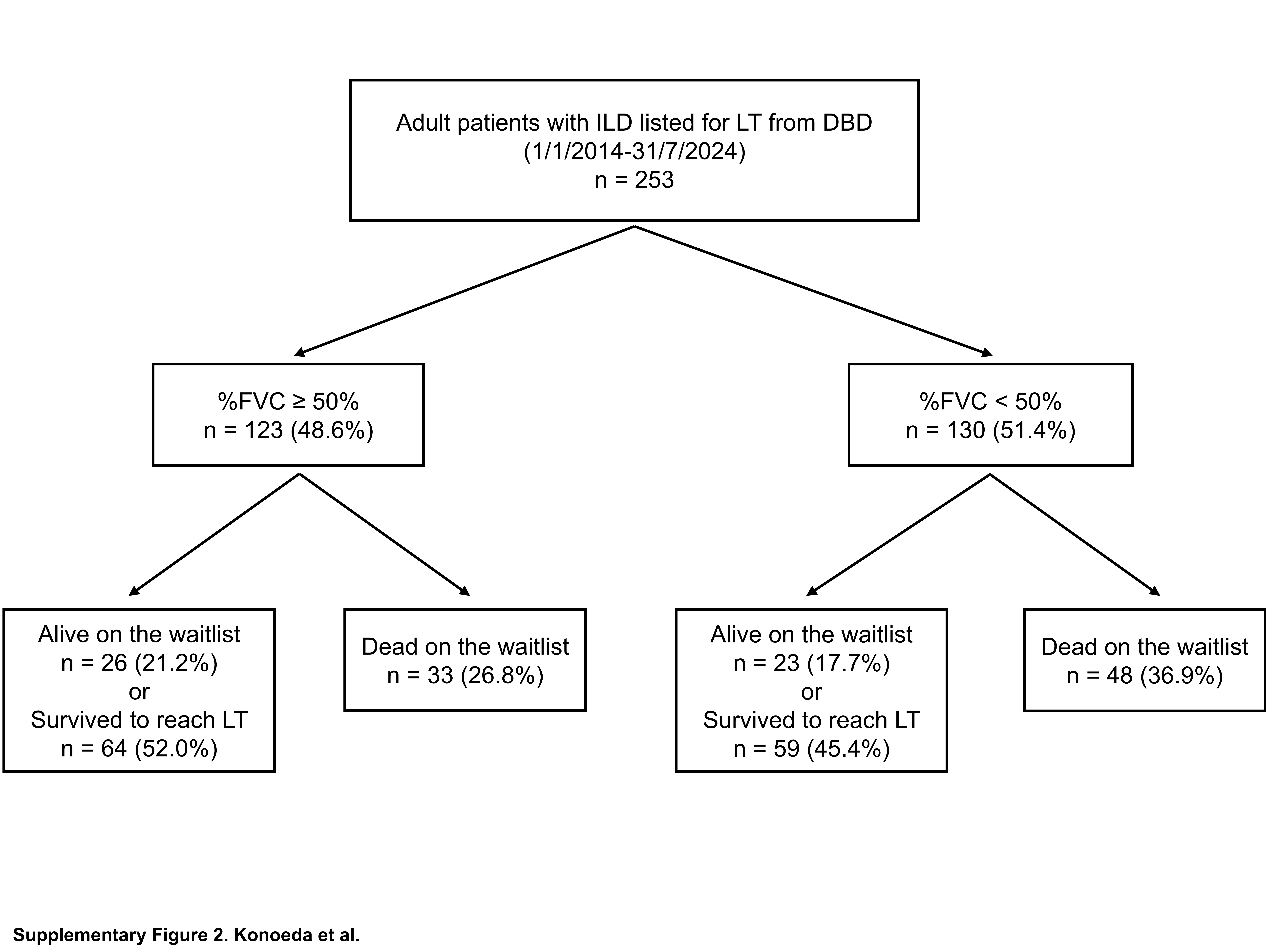

Supplement: Supplementary file 3 — Supplementary Figure 2. Study flowchart. The analyzed population comprised 253 adult patients with ILD listed for LT from DBD according to %FVC. DBD, donation after brain death; ILD, interstitial lung disease; LT, lung transplantation; %FVC, percentage forced vital capacity [file 11748_2026_2273_MOESM3_ESM.tif]

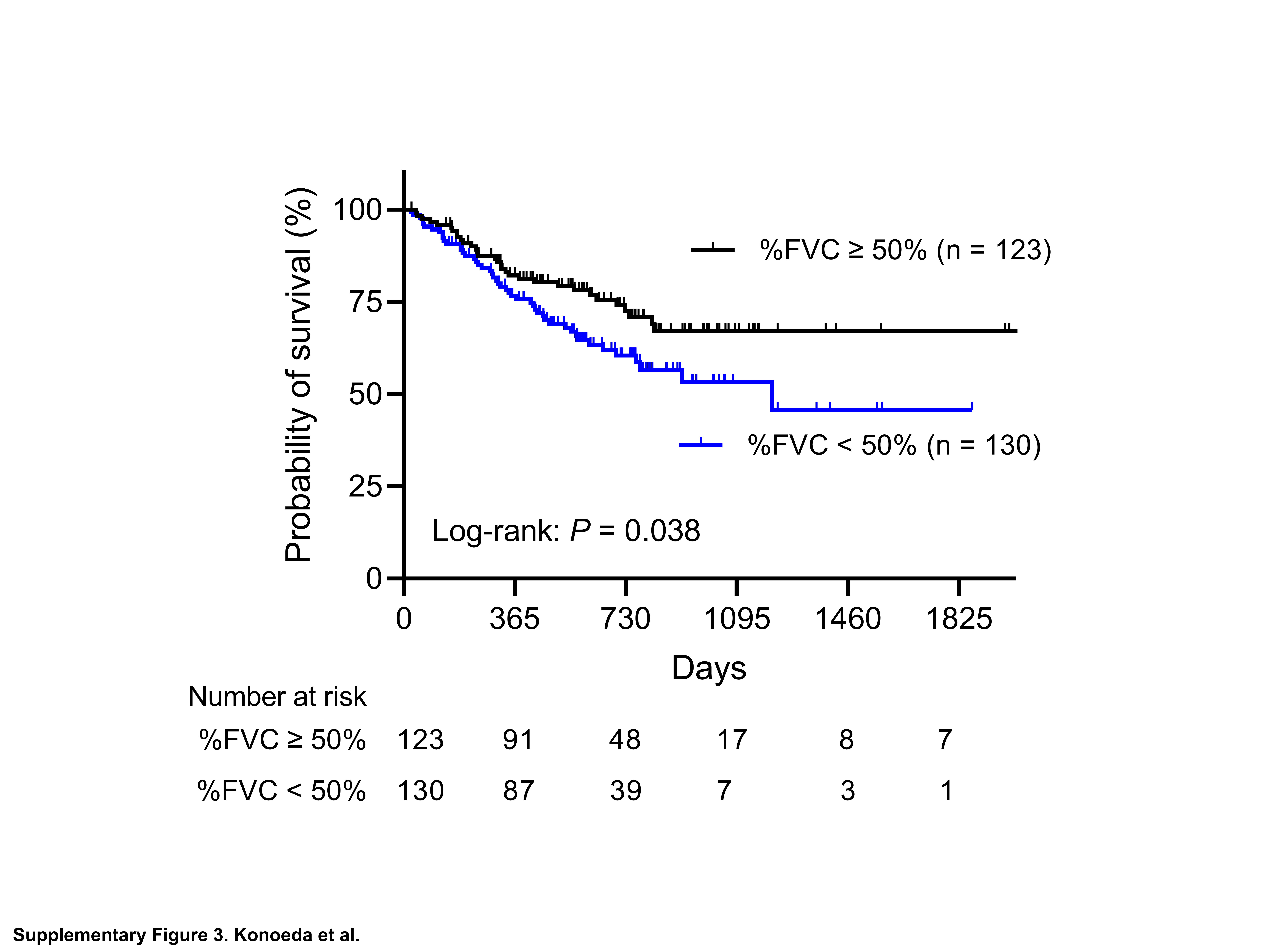

Supplement: Supplementary file 4 — Supplementary Figure 3. Waitlist mortality in 123 patients with %FVC ≥ 50% and 130 patients with %FVC < 50% listed for LT. The %FVC ≥ 50% group versus %FVC < 50%; log-rank: P = 0.038. LT, lung transplantation; %FVC, percentage forced vital capacity [file 11748_2026_2273_MOESM4_ESM.tif]

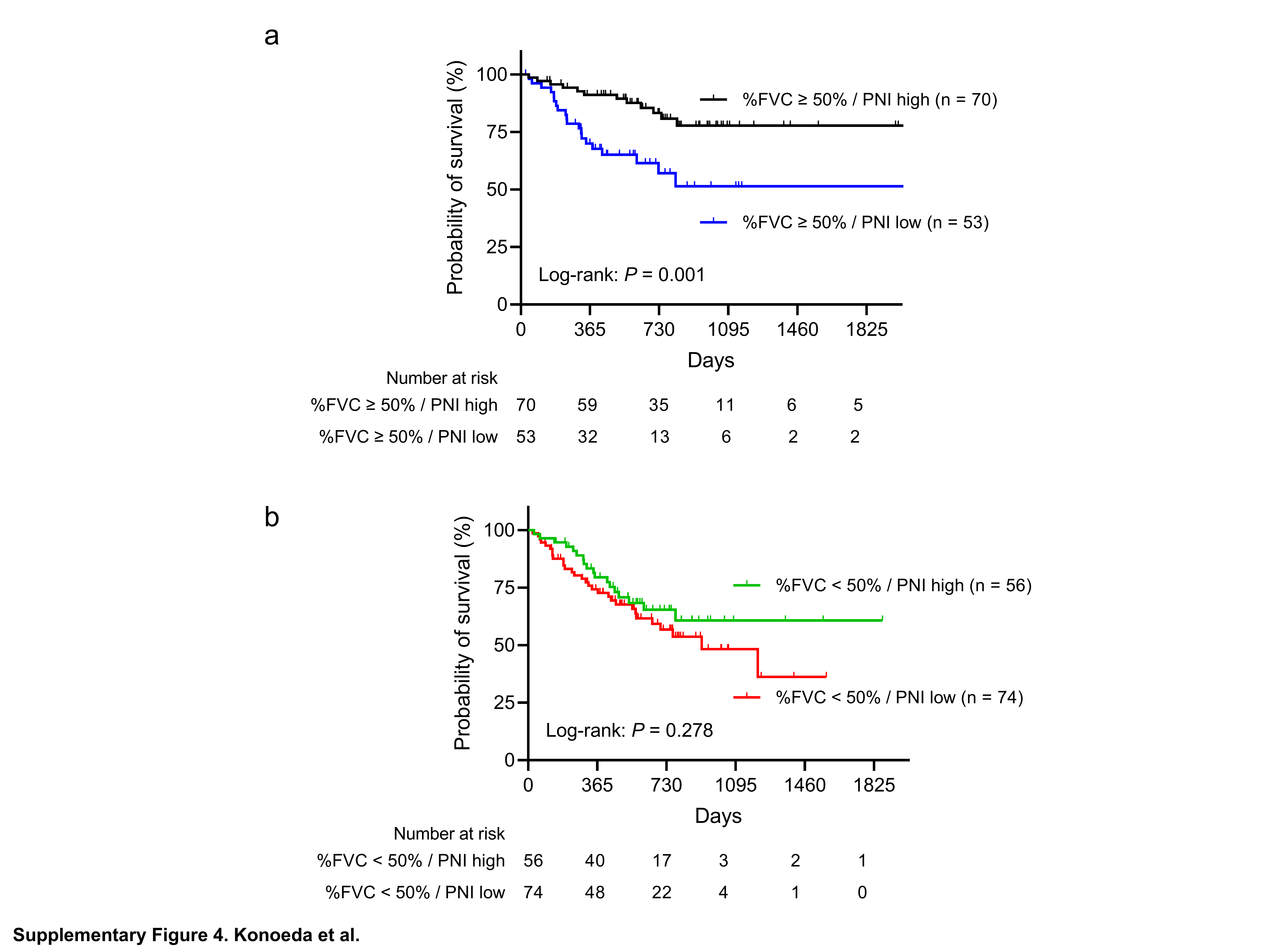

Supplement: Supplementary file 5 — Waitlist mortality in 123 patients with %FVC ≥ 50% and 130 patients with %FVC < 50% listed for LT based on their PNI. (a) The %FVC ≥ 50% group: low PNI (< 45.8; n = 53) versus high PNI (≥ 45.8; n = 70); log-rank: P = 0.001. (b) The %FVC < 50% group: low PNI (< 45.8; n = 74) versus high PNI (≥ 45.8; n = 56); log-rank: P = 0.278. LT, lung transplantation; %FVC, percentage forced vital capacity; PNI, prognostic nutritional index [file 11748_2026_2273_MOESM5_ESM.tif]
